# Condition-dependent success of resource-based reproductive tactics across both sexes in *Drosophila prolongata*

**DOI:** 10.1007/s00265-026-03739-z

**Published:** 2026-05-07

**Authors:** Alessio N. De Nardo, Swarnima Mukherjee, Stefan Lüpold

**Affiliations:** 1 Department of Evolutionary Biology and Environmental Studies, University of Zurich, Zurich, Switzerland; 2 Department of Integrative Biology, Michigan State University, East Lansing, MI USA

**Keywords:** Sexual selection, Alternative reproductive tactics, Scramble competition, Interception, Aggression, Courtship, Territory

## Abstract

Because reproductive tactics vary in their energetic and fitness costs, individuals may increase their reproductive success by aligning their tactics with their physical condition. In resource-based mating systems, monopolizing valuable patches is energetically costly, yet potentially highly rewarding for males. In females, mating near resources may reduce the cost of mate search and assessment. However, the balance of costs and benefits associated with a given tactic may shift with an individual’s condition. Here we studied this in the fruit fly *Drosophila prolongata*. In arenas containing resource-dense areas (RDAs) typically used as territories, we tracked the individual positions and behaviors of flies in groups using machine-learning-based pose estimation and behavioral classification. We found that large, high-condition males achieved elevated mating success through territoriality and courtship, whilst small males obtained copulations through high mobility, consistent with a scramble competition tactic. Female behavior was also condition dependent: large females spent more time in RDAs, mated sooner at those sites, and thus reinforced territory-based sexual selection, whereas small females mated later and more frequently outside RDAs, potentially avoiding harassment costs. In addition, we detected size-disassortative pairings, suggesting that the complex cost-benefit interactions encountered by females of varying condition can lead to counter-intuitive associations. However, given the limited duration of our observations and the high remating rate in this species, the prevalence of this disassortative mating pattern over longer time scales warrants further investigation. Our findings reveal how resource distribution and individual condition may interact to maintain multiple reproductive tactics in both sexes.

**Supplementary information:**

The online version contains supplementary material available at https://doi.org/10.1007/s00265-026-03739-z.

## Introduction

Sexual selection significantly shapes the evolution of elaborate traits and behaviors that mediate mate acquisition and reproductive success (Andersson and Simmons 2006). Because it is typically stronger on males than on females (Andersson 1994; Janicke et al. 2016), it can drive male reproductive strategies and mating behaviors. The specific tactic used by an individual to maximize reproductive success can depend on the prevailing social and environmental context (e.g., Emlen and Oring 1977; Gwynne and Simmons 1990; McLain 1992; Mills and Reynolds 2003; Reichard et al. 2004; Buzzatto et al. 2014). For example, in the European bitterling (*Rhodeus sericeus*), large males can monopolize females and achieve nearly exclusive fertilizations at low competitor densities, but they lose fertilization opportunities to small males as higher densities (Mills and Reynolds 2003).

Any uneven distribution of resources such as food, oviposition sites or potential mates, allows competitively superior males to monopolize them (Brown 1964; Emlen and Oring 1977; Grant 1993), linking reproductive success to resource defense or territoriality (Thornhill and Alcock 1983; Shuster and Wade 2003). However, territorial defense can be costly through heightened risks of injury or mortality, elevated energetic expenditure, or reduced foraging time (reviewed in Ord 2021). Thus, territoriality is only favored when its fitness benefits exceed these costs (Brown 1964; Grant 1993). Additionally, not all territories are equally defensible; for instance, when territory size or an individual’s condition renders defense too costly (Brown 1964; Grant 1993).

When resources are difficult to monopolize, some males, particularly those in poor condition, may adopt an alternative reproductive tactic (ART), such as remaining near resources or territories that attract females and securing mating opportunities through non-territorial behavior around monopolizing males (Kodric-Brown 1986; Gosling and Petrie 1990; Gross 1996; Shuster and Wade 2003; Mills and Reynolds 2003; Berec and Bajgar 2011; York et al. 2014; Ramírez-Delgado et al. 2015; Zarini et al. 2023). These tactics can yield reproductive success comparable to that of territorial males (Da Silva et al. 2025). For example, in the cichlid *Lamprologus callipterus*, large males aggressively defend breeding sites, whilst smaller males follow a scramble tactics to exploit brief spawning opportunities (Von Kuerthy and Taborsky 2016). Similarly, males of the American rubyspot damselfly (*Hetaerina americana*) adopt territorial, non-territorial, or flexible strategies based on their thoracic fat reserves (Raihani et al. 2008).

Male tactic expression can be fixed by phenotype (e.g., size) or flexible in response to socio-ecological factors (e.g., competitor density, resource distribution; Herberstein et al. 2017). When resources or females become widely dispersed, or when male densities hamper effective resource defense, many males may switch from territorial behavior to scramble competition, a flexible alternative reproductive tactic that favors rapid mate search over aggression (Knell 2009; Herberstein et al. 2017). For example in the seed bug *Neacoryphus bicrucis*, most large males abandon patch monopolization at high densities, and body size becomes less relevant under scramble competition (McLain 1992). The threshold for switching from resource defense to a scramble tactic depends on a male’s condition and the expected fitness returns with either strategy (Dubois et al. 2003; Herberstein et al. 2017), and such flexible shifts to an alternative tactic often come with fitness costs (Da Silva et al. 2025).

But why should females choose territorial males in the first place? After all, female mate choice comes with its own costs, including search and sampling effort (Parker 1983; Byers et al. 2005), predation risk (Lima and Dill 1990; deRivera et al. 2003; Kim et al. 2007; Bonachea and Ryan 2011; Edomwande and Barbosa 2020), or the potential loss of high-quality mates to faster-choosing females (Dale et al. 1992; Lindström and Lehtonen 2013). The benefits of choosiness must outweigh these costs for non-random choice to be selected (Pomiankowski 1987).

In some species, the direct benefits of choosing a male with a high-quality territory are evident: the area may provide abundant food, a good breeding site, and/or protection from predators (Verner and Willson 1966; Carranza et al. 1990; Hoffmann and Cacoyianni 1990; Calsbeek and Sinervo 2002; Dijkstra et al. 2008; Verdolin and Slobodchikoff 2010; Tregenza et al. 2022). Additionally, choosing a territorial male may indirectly select for high mate quality, potentially genetically enhancing offspring fitness (Candolin and Voigt 2001; Seddon et al. 2004; Vanpé et al. 2007; White and Rundle 2015), or simply facilitate mate searching and choice due to their site fidelity, particularly if territory size or quality is correlated with male quality (Candolin and Voigt 2001; Deb and Balakrishnan 2014). This would allow for indirect mate choice (also referred to as ‘passive’ or ‘opportunistic’; Wiley and Poston 1996; Castellano 2009; Kovalov and Kokko 2022), avoiding the cost of active sampling or rejection (see Kokko et al. 1999 for analogous arguments for lekking species).

Like males, females vary in their condition (e.g., size) and in their vulnerability to environmental or social stressors. For example, the fitness of low-quality *Drosophila melanogaster* females is affected more by male harassment than that of their higher-quality counterparts (MacPherson et al. 2018). Similarly, females may benefit from adjusting their mate preferences or acceptance thresholds depending on their body condition (Frame and Mills 2014), such as in the black field cricket, *Teleogryllus commodus*, where females lower their preference threshold for male calls if they have been raised on a low-quality diet (Hunt et al. 2005). Thus, the relative cost of choice or harassment may vary with female condition (Jennions and Petrie 1997; but see Dougherty 2023). If so, female preference for territorial over non-territorial males could conceivably also be condition-dependent, generally being stronger for high- compared to low-condition females.

Here, we investigated the effect of resource defense on mating success and how it depends on male condition, as well as female condition-dependent mating decisions in *Drosophila prolongata.* This South-Asian fruit fly is unique among *Drosophila* for its conspicuous male-biased sexual dimorphism in body size and in foreleg size and coloration (Setoguchi et al. 2014; Rohner et al. 2018). Males are highly aggressive and show territorial behavior on high-quality resources (Kudo et al. 2015; Amino and Matsuo 2020, 2023; De Nardo et al. 2025a). Whilst territoriality increases mating success under an even sex ratio (De Nardo et al. 2025a), a male-biased sex ratio favors scramble competition and smaller body size (De Nardo et al. 2025a; Nardo et al. 2025b). Males exhibit elaborate courtship behavior using their enlarged forelegs (Setoguchi et al. 2014), but low-condition males often adopt an alternative mating tactic by intercepting rivals’ mating attempts (Perdigón Ferreira and Lüpold 2022).

In this experiment, we recorded groups of flies in arenas with two resource-dense areas (RDAs) differing in size and quality. Using machine-learning approaches, we tracked individual flies and predicted their behaviors. In a first account, based on data from the same experiment, we studied how males and females distribute in and around RDAs (Mukherjee et al. 2026). We found that both males and females have a strong preference for large RDAs, while quality was secondary. However, large RDAs were more difficult to monopolize as they attracted more competitors and so needed more aggressive interactions to secure occupancy. For females, time spent on resources increased during and after mating, indicating a preference for these areas for both oviposition and mate searching.

In the present study, we focus on the effect of resource occupancy on reproductive success, with special attention to condition-dependent reproductive strategies in both sexes. We predicted that (i) large males are more aggressive and more successful at monopolizing resources; (ii) this leads to higher mating success for large males; (iii) small males are more likely to adopt non-territorial mating tactics (i.e., are more mobile); (iv) courtship increases mating success, but its effect is modulated by body size; and (v) large females exhibit a stronger preference for large, territorial males, whereas small females mate more opportunistically within territories, potentially reducing the costs of active mate choice.

## Materials and methods

### Study organisms

We used flies derived from a genetically diverse population originally collected in Vietnam and maintained in laboratory conditions for about 50 generations (20 ± 1 °C, 60% relative humidity, 14:10 light: dark cycle). To generate body size variation in the experimental flies, we reared them at controlled densities of 20 eggs per vial (60mL vial, containing 12mL of food) at three different food qualities (high, intermediate, low). High-quality food is the standard food in the lab (55 g corn, 100 g yeast, 75 g glucose, 8 g agar, 10 g flour, 15mL Nipagin per liter), while intermediate and low-quality food was achieved by diluting this medium with water and agar by ½ or ¼, respectively. We separated males and females within 24 h of eclosion to ensure virginity, keeping individuals in groups of 5 males or 15 females.

### Experimental setup

To identify individuals within experimental arenas, we marked them with a unique color dot (Kreul El Greco Acrylic colors) on their thorax under ice anesthesia at least two days before the experiment. Flies remained in isolation between marking and the start of the experiment. We then placed groups of eight males and eight females in Petri dishes (ø10 cm), containing a large and a small resource-dense area (RDA; Fig. S1). The large RDA was defined by a square plastic container (2.2 ⋅ 2.2 ⋅ 0.8 cm; approx. 7.5% of total area), whilst the small RDA was created by elevating the cap of a 5mL Eppendorf tube (ø 0.9 cm; approx. 1.3% of total area) using putty, matching the height of the large RDA. Both RDAs were filled to the edge with standard fly medium (see above) and the peripheral Petri dish was filled with orange juice agar (600mL water, 200mL orange juice, 18 g agar, 20 g saccharose, 20mL Nipagin per approx. 800mL) up to the same level. To vary resource quality in addition to resource size, we either covered the RDAs with yeast paste (high quality; a mixture of dry yeast and orange juice, preferred oviposition substrate) or sprinkled with dry yeast alone (lower quality, but still suitable oviposition substrate). Each arena contained a large and a small RDA, whereas quality treatments varied in a full-factorial design (i.e. 4 types of treatment: both RDAs high quality, both RDAs low quality, large RDA high and small RDA low, large RDA low and small RDA high). In contrast to the RDAs, the peripheral orange-juice agar provided carbohydrates for the adults but constituted a low-quality oviposition substrate, thereby encouraging oviposition within the RDAs.

We recorded all groups of 16 flies in 10 replicate arenas for each of the four RDA treatments using eight Raspberry Pi camera modules (V2) mounted above the setup, inside a climate chamber at 20 ± 1 °C and 60% relative humidity. Flies from different developmental treatments were haphazardly assigned to arenas, as the goal was not to test for developmental diet effects but to generate random body size variation within units (Fig. S2). We imaged arenas every two seconds for > 30 min either between 10:00 and 12:00 (2–4 h after lights on) or between 14:00 and 16:00 (6–8 h after lights on). During each session, we recorded two arenas per RDA treatment across eight cameras, balancing the orientation between replicate arenas (i.e., large RDA once facing left and once right) to control for positional bias. All arenas were recorded within a five-day period. Across the 40 arenas, the total sample size was 320 individuals per sex, but one male escaped during recording and so was excluded from downstream analysis due to incomplete data and lacking body size measurement.

### Processing of recordings, fly tracking and behavioral categorization

We transformed the first 30 min of the captured images to mp4 video format and trained the deep-learning software SLEAP version 1.3.0 (Social LEAP Estimates Animal Poses; Pereira et al. 2022) to track individuals and their body parts. Detailed protocols and explanations are provided in (Mukherjee et al. 2026). In each recording, the two RDAs were marked as regions of interest (ROIs) using Fiji (Schindelin et al. 2012). As the probability of being randomly located in the large RDA was much higher due to its size, a circular control area or buffer zone was added around the center of the small area, covering the same total area as the large RDA (Fig. S1; also approx. 7.5% of total area). In contrast to Mukherjee et al. (2026), where the small RDA was analyzed either alone or with the buffer zone depending on the analysis, here we always include the buffer zone when referencing the small RDA.

Utilizing tracking data from SLEAP, we derived information regarding the positions and postures of each individual, along with their spatial relationships relative to other individuals. These data were used to determine whether individuals were within an RDA (i.e., their thorax coordinates fell inside the defined ROI) and to train a random forest classifier for identifying matings, courtship and aggression in males, using the Python library *scikit-learn* (Pedregosa et al. 2011; Varoquaux et al. 2015). Details on the features used, training protocols and post-prediction corrections are described in detail in Mukherjee et al. (2026). Using this approach and after minimal post-prediction correction we reached high precision and recall for mating events (precision: 1.0, recall: 0.979) and accuracies of 98.5% and 98.6% for aggression and courtship, respectively (Mukherjee et al. 2026).

#### Variable definitions

All variables and behaviors are defined in Table 1, along with a description of their quantification.

**Table 1:** Definitions and quantification of behaviors and variables used in the analysis [(partially overlapping with Table 1 in (Mukherjee et al. 2026)]

Variable / Behavior	Definition	Quantification
Occupancy	*Behavior*: Whether an individual is located in an RDA *Variable*: Occupancy as used in the analyses is the proportion of frames an individual occupies an RDA in a given period (e.g., overall, during non-mating or as a virgin). We excluded the time *in copula* from measures of occupancy, except where stated explicitly, following (Mukherjee et al. 2026)	*Behavior*: Thorax coordinate inside of predefined region of interest in that frame *Variable*: Proportion of occupancy frames out of total observed frames. In this study, the frames during which an individual was *in copula* (i.e., detected as mating frame) were excluded from the occupancy calculation.
Mobility	*Per frame*: Distance moved since previous frame *Variable*: Medina distance moved between frames within given period (e.g., overall or per minute)	*Per frame*: Vector length of the frame-to-frame displacement in the x- and y-coordinates of the thorax (or head if thorax is not available) between frames in pixels *Variable*: Median vector length within given period
Aggression	*Behavior*: Established agonistic behaviors between individual *Drosophila* males, including chasing, fencing, lunging, tussling and boxing, not including threats (e.g., Chen et al. 2002; Jonsson et al. 2011; Zwarts et al. 2012; Belenioti and Chaniotakis 2020). Ethogram in Table S1. All of these were considered aggressive behavior. *Variable*: Proportion of frames in which aggressive behavior was observed out of total observed frames in a given period (i.e., aggression rate)	*Behavior*: Manual labeling of training data frames following the ethogram (Table S1) for behavioral categorization by random forest classifier. Trained model predicts instances of aggressive behavior on frame by frame level for each male. We followed the ethogram to decide whether a behavior is aggressive, but did not differentiate the different types of aggressive behaviors for training. *Variable*: Proportion of frames for which aggressive behavior was predicted by random forest classifier out of total observed frames in a given period (i.e., aggression rate)
Courtship	*Behavior*: Established courtship behaviors between *Drosophila prolongta* males and females, including following, licking, elbow rubbing, wing vibration, wing waving, leg display, leg vibration etc. (Setoguchi et al. 2014). *Variable*: Proportion of frames in which courtship behavior was observed out of total observed frames in a given period (i.e., courtship rate)	*Behavior*: Manual labeling of training data frames following the ethogram in Setoguchi et al. (2014) for behavioral categorization by random forest classifier. Trained model predicts instances of courtship behavior on frame by frame level for each male. We did not differentiate the different types of aggressive behaviors for training. *Variable*: Proportion of frames for which courtship was predicted by random forest classifier out of total observed frames in a given period (i.e., courtship rate)

### Statistical analysis

The data was analyzed and visualized in R version 4.3.1 (R Core Team 2023). We fitted linear mixed effects models (LMM) to group-standardized variables, with arena as a random effect, using *lme4* (Bates et al. 2015). Mating success was analyzed separately using generalized linear mixed effects models (GLMM) with a binomial error structure. Focal and relevant control variables were retained in all models, but interactions included to test for condition-dependent effects were sequentially removed from full models if their *P >* 0.1 and their exclusion improved model fit. These omitted interactions are not reported in *Results*.

Although all our primary analyses were based on standardized thorax length, we additionally dichotomized males by their size using a *k*-means clustering approach to conduct separate analyses for ‘small’ and ‘large’ males within units. This approach was particularly useful for comparing size-specific pathways in structural equation models, as interactions with continuous size cannot be readily incorporated into these models. Clustering was based on the 25th and 75th percentiles of thorax length, which served as the centers for ‘small’ and ‘large’ male categories, respectively, resulting in non-overlapping distributions of body sizes within each of the clusters (Fig. S3). Due to the haphazard assignment of flies to units, the number of individuals categorized as small or large varied between units but did not systematically differ between treatments. Each male was classified based on its thorax length relative to these cluster centers and the distribution of other males in the same arena, resulting in 125 ‘small’ and 194 ‘large’ males.

Model assumptions were checked using *DHARMa* (Hartig 2016), whilst model predictions were made using *ggeffects* (Lüdecke 2018) and visualized using *ggplot2* (Wickham 2016). Structural equation modeling (SEM) was done using *piecewiseSEM* (Lefcheck 2016), with arenas as random effects, visualizing results using *DiagrammeR* (Iannone 2023). Finally, we used *mediation* (Tingley et al. 2014) for mediation models and *emmeans* (Lenth 2023) for pairwise comparisons.

## Results

### Developmental food treatments

Body size as measured by thorax length was similarly affected by larval diet in each sex (*X*^2^_2_ = 4.93, *n* = 639, *P =* 0.085; Fig. S4; Table S2), being larger after developing in high-quality compared to intermediate (females: *t* = -4.68, *P* < 0.001; males: *t* = -2.76, *P* < 0.01) or low-quality food (females: *t* = -8.87, *P* < 0.001; males: *t* = -10.71, *P* < 0.001), and if developing in intermediate compared to low-quality food (females: *t* = -4.99, *P* < 0.001; males: *t* = -8.37, *P* < 0.001). However, due to considerable overlap in body size between food treatments (Fig. S4), analyses included thorax length as a measure of body size instead of food treatments.

### Male mating behavior and mating success

#### Across all males

Across *n* = 319 males, overall aggression increased with thorax length (*t* = 2.13, *P* = 0.034). Aggression (*t* = 9.11, *P <* 0.001; Table S3), but not thorax length (*t* = -0.23, *P* = 0.82), also covaried positively with occupancy of the large RDA (occupancy-L). Accounting for occupancy-L (*t* = -7.76, *P* < 0.001; Table S4), however, neither aggression (*t* = 1.48, *P* = 0.14) nor thorax length (*t* = -0.92, *P* = 0.36) affected occupancy of the small RDA (occupancy-S).

On average, males spent 20.1 ± 1.1% (mean ± S.D.) of the total time on aggression, i.e., 6 min and 2 seconds out of the 30-minute observation period, and 57.0% of that time was observed outside of RDAs, which was less than expected by chance (85%; *t* = -25.33, *P* < 0.001). By contrast, aggression was disproportionately frequent in both the small (9.3%; *t* = 2.72, *P* = 0.007) and large RDAs (33.7%; *t* = 18.51, *P* < 0.001; expected by chance: 7.5% each; Fig. S5a). Relative to the time spent in each of the three areas, agonistic interactions constituted a smaller percentage outside RDAs (17.6%) than they did in either the small (28.8%; *t =* 6.82, *P* < 0.001) or the large RDA (34.4%; *t* = 10.94, *P <* 0.001; Fig. S5b), respectively. Lastly, while aggression was frequently observed outside RDAs, males with high occupancy-L expressed very little aggression outside RDAs, but slightly more if they also had high occupancy-S (*X*^2^_1_ = 5.96, *P =* 0.015; Table S5; Fig. S5c).

Furthermore, males spent 21.1 ± 1.3% of the total time courting, i.e., 6 min and 20 s out of the 30-minute total. The accumulated courtship period increased with occupancy of either RDA (both *t* ≥ 2.97, *P* ≤ 0.003), albeit more so with occupancy-L for larger males (*X*^2^_1_ = 5.94, *P =* 0.015; Fig. S7; Table S6). Thorax length had no main effect (*t* = 0.76, *P* = 0.45).

Of all courtship, males expressed the greatest proportion outside of RDAs (62.9%), but less than expected by chance (85%; *t* = -18.15, *P* < 0.001), whilst courtship exceeded the 7.5% predicted by occupancy in both the small (9.0%; *t* = 2.10, *P* = 0.036) and the large RDA (28%; *t* = 14.65, *P* < 0.001; Fig. S6a). Consistently, the relative time spent courting within each area was higher in the small (27.5%; *t* = 4.32, *P* < 0.001) and large RDA (27.4; *t* = 4.56, *P* < 0.001) compared to the periphery (20.1%; Fig. S6b). As with aggression, males often courted outside RDAs overall, but those with high occupancy-L did so rarely and primarily when they also had high occupancy-S (*X*^2^_1_ = 9.7, *P =* 0.002; Fig. S6c).

The mean distance that males moved between frames (i.e., mobility) increased with courtship (*t* = 17.97, *P* < 0.001; Table S7) and aggressive behavior, with the latter effect being stronger in large males (*X*^2^_1_ = 5.87, *P =* 0.016). However, males with higher occupancy-L had lower mobility (*t* = -2.97, *P* = 0.046), while occupancy-S had no effect (*t* = -1.21, *P* = 0.23).

Overall, courtship enhanced relative male mating success (*t* = 2.56, *P* = 0.010; Table S8), while the effect of aggression was size-dependent: a mediation model indicated that aggression positively affected relative mating success indirectly by increasing occupancy-L (*P* = 0.027, *n*_*sim*_ = 2000). However, the direct effect of aggression on mating success (beyond the effect on occupancy) showed an interaction with male size: it shifted from slightly positive to negative as thorax length increased (*X*^2^_1_ = 4.02, *P =* 0.045; Fig. S8). In contrast, relatively large males gained mating success by occupying RDAs, especially when using both types, whilst RDA occupancy had no effect on relative mating success for smaller males (*X*^2^_1_ = 7.15, *P =* 0.007; Fig. S9). Finally, while controlling for courtship and aggressive behavior that correlated with it, mobility still increased with relative mating success (*t* = 2.00, *P* = 0.047).

A piecewise SEM across all males (Fig. S10; Table 2) revealed that courtship (standardized estimate *β* = 0.25, *P* = 0.003) and occupancy-L (*β* = 0.14, *P* = 0.035) directly increased relative mating success, while courtship itself was positively affected by both occupancy-L (*β* = 0.32, *P* < 0.001) and -S (*β* = 0.17, *P* = 0.004). Occupancy-L was increased by aggression (*β* = 0.46, *P* < 0.001), which in turn increased with thorax length (v = 0.12, *P* = 0.034). Occupancy-S was negatively affected by occupancy-L (*β* = -0.45, *P* < 0.001). Lastly, mobility increased with the frequency of courtship (*β* = 0.65, *P* < 0.001) and aggression (*β* = 0.35, *P* < 0.001) but decreased with occupancy-L (*β* = -0.12, *P* = 0.003).

**Table 2:** Standard estimates (Std. est.) and *P*-values of structural equation models (SEM) across all males, large males or small males, respectively. Significant estimates are indicated in bold (*P* < 0.05) and trends in italics (0.05 < *P* < 0.1).

		Across all males(*n*= 319)		Large males(*n*= 194)		Small males(*n*= 125)	
Response	Predictor	Std. est.	*P*	Std. est.	*P*	Std. est.	*P*
Aggression	Body size	**0.12**	**0.033**	-	-	-	-
Occupancy-L	Body size	-0.01	0.82	-	-	-	-
	Aggression	**0.46**	**< 0.001**	**0.47**	**< 0.001**	**0.43**	**< 0.001**
Occupancy-S	Body size	-0.05	0.36	-	-	-	-
	Aggression	0.09	0.14	-0.00	0.95	0.21	0.024
	Occupancy-L	**-0.45**	**< 0.001**	**-0.45**	**< 0.001**	**-0.44**	**< 0.001**
Courtship	Body size	0.02	0.67	-	-	-	-
	Occupancy-L	**0.32**	**< 0.001**	**0.38**	**< 0.001**	0.21	**0.026**
	Occupancy-S	0.17	0.004	0.15	0.046	0.21	**0.026**
Mobility	Body size	0.05	0.11	-	-	-	-
	Courtship	**0.64**	**< 0.001**	**0.63**	**< 0.001**	**0.66**	**< 0.001**
	Aggression	**0.35**	**< 0.001**	**0.39**	**< 0.001**	**0.33**	**< 0.001**
	Occupancy-L	**-0.11**	**0.003**	*-0.09*	*0.056*	**-0.16**	**0.020**
	Occupancy-S	-0.5	0.14	-0.01	0.82	-0.09	0.16
Relative matingsuccess	Body size	0.08	0.15	-	-	-	-
	Aggression	-0.05	0.52	-0.13	0.19	0.12	0.26
	Courtship	**0.24**	**0.004**	**0.34**	0.003	0.04	0.76
	Occupancy-L	**0.14**	**0.032**	**0.18**	**0.039**	0.08	0.42
	Occupancy-S	0.07	0.24	0.10	0.21	-0.00	0.96
	Mobility	*0.16*	*0.088*	0.06	0.64	**0.41**	**0.002**

#### Large males

Focusing only on the males categorized as ‘large’, constituting 60.8% of all males that gained 67.2% of all matings (156 out of 232), relative mating success increased with courtship (*t* = 2.64, *P* = 0.009; Table S9) and the occupancy of both large and small RDAs, especially in combination (*X*^2^_1_ = 4.08, *P =* 0.043). Thorax length had no effect on relative mating success (*t* = -0.25, *P* = 0.80), nor did aggression (*t* = -1.44, *P* = 0.15) or mobility (*t* = 0.67, *P* = 0.50).

**Fig. 1:**
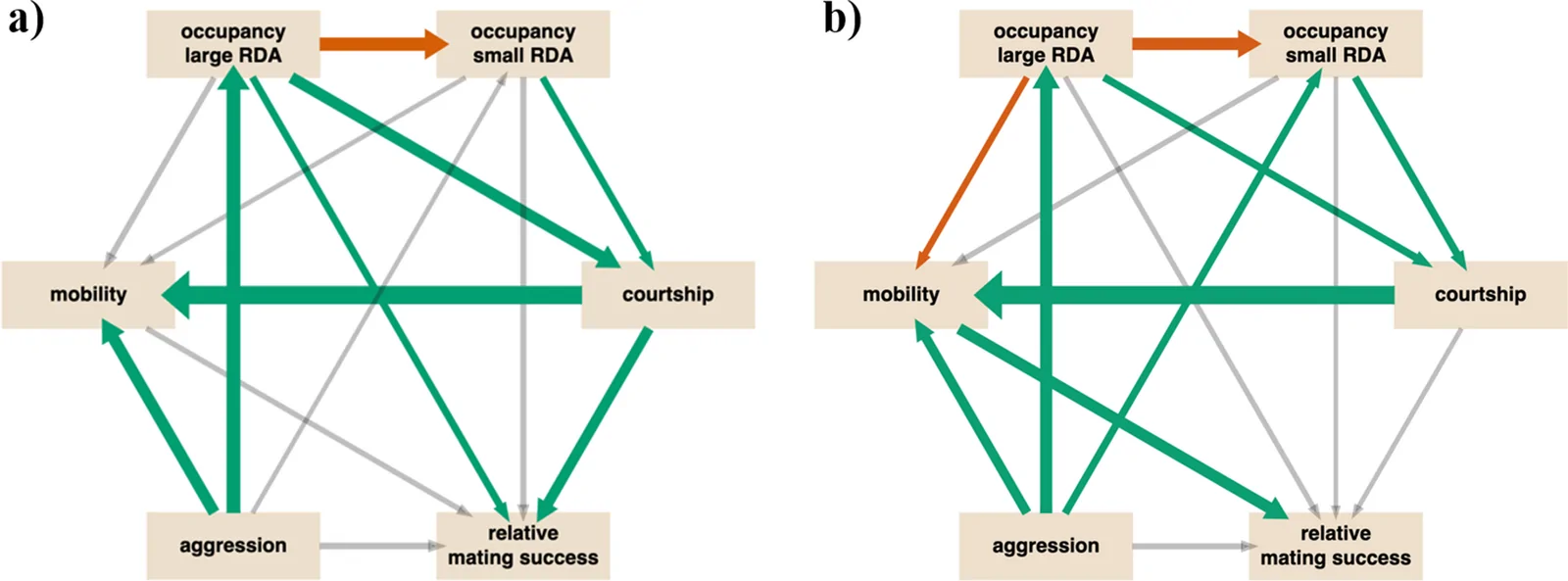
Visualization of structural equation models across (**a**) large males and (**b**) small males. Green arrows indicate significantly positive effects, red arrows indicate significantly negative effects, while grey arrows indicate hypothesized effects with *P* > 0.05. The width of the arrow reflects the effect size

As across all males, the SEM on large males indicated that relative mating success increased with occupancy-L (*n* = 194; *β* = 0.18, *P* = 0.039; Fig. 1a; Table 2) and total courtship duration (*β* = 0.34, *P* = 0.003). More aggressive males also had higher occupancy-L (*β* = 0.47, *P* < 0.001), whilst occupancy-S was negatively affected by occupancy-L (*β* = -0.45, *P* < 0.001) and courtship increased with both occupancy-S (*β* = 0.15, *P* = 0.046) and -S (*β* = 0.38, *P* < 0.001), and mobility increased with courtship (*β* = 0.63, *P* < 0.001) and aggression (*β* = 0.39, *P* < 0.001).

### Small males

Males classified as ‘small’ represented 39.2% of all males and achieved 32.8% of all matings (76 out of 232). Among these males, neither courtship (*t* = 0.45, *P* = 0.65; Table S10) nor occupancy-S (*t* = 0.16, *P* = 0.88) or -L (*t* = 0.79, *P* = 0.43) affected relative mating success. As in large males, there was no effect of aggression (*t* = 0.77, *P* = 0.44), but mating success tended to increase with thorax length (*t* = 1.90, *P* = 0.06). However, unlike large males, high mobility enhanced the probability of higher mating success (*t* = 3.14, *P* = 0.002), controlling for courtship and aggression.

The SEM for small males (*n* = 125; Fig. 1b; Table 2) identified mobility as the only focal predictor of relative mating success (*β* = 0.41, *P* = 0.002). As in large males, aggression increased both occupancy-L (*β* = 0.43, *P* < 0.001) and occupancy-S (*β* = 0.21, *P* = 0.024) and occupancy-L in turn reduced occupancy-S (*β* = -0.44, *P* < 0.001).

Courtship was explained by the occupancy of both the large (*β* = 0.21, *P* = 0.026) and the small RDAs (*β* = 0.21, *P* = 0.026). Mobility increased with both courtship (*β* = 0.66, *P* < 0.001) and aggression (*β* = 0.33, *P* < 0.001) but decreased with occupancy-L (*β* = -0.16, *P* = 0.020).

### Female mating behavior and mating success

Female mating status was coded as a binary variable (mated [1], not mated [0]), as only three out of the 320 females mated twice. A binomial GLMM revealed that females were more likely to mate when they spent more time in the large RDA (*z* = 2.83, *n =* 320, *P* = 0.005; Table S11). Time spent on the small RDA (*z* = 1.11, *P* = 0.27), thorax length (*z* = 0.73, *P* = 0.46) and mobility (*z* = 1.01, *P* = 0.31) had no significant effect. Time spent on large RDAs increased with female body size (*t* = 2.51, *P* = 0.013; Table S12) and their mobility before their first mating (*t* = 3.46, *P* < 0.001), neither of which was the case for small RDAs (body size: *t* = 0.45, *P* = 0.65; mobility: *t* = 1.57, *P* = 0.12; Table S13). Time on the small RDA itself declined with time spent on its large counterpart (*t* = -4.18, *P* < 0.001). A piecewise SEM combining these models confirmed these effects (Table S14; Fig. S11).

In mated females, thorax length did not affect time spent on large (*t* = 1.27, *P* = 0.21) or small RDAs (*t* = 0.23, *P* = 0.82).

### Mating-level analysis

**Fig. 2:**
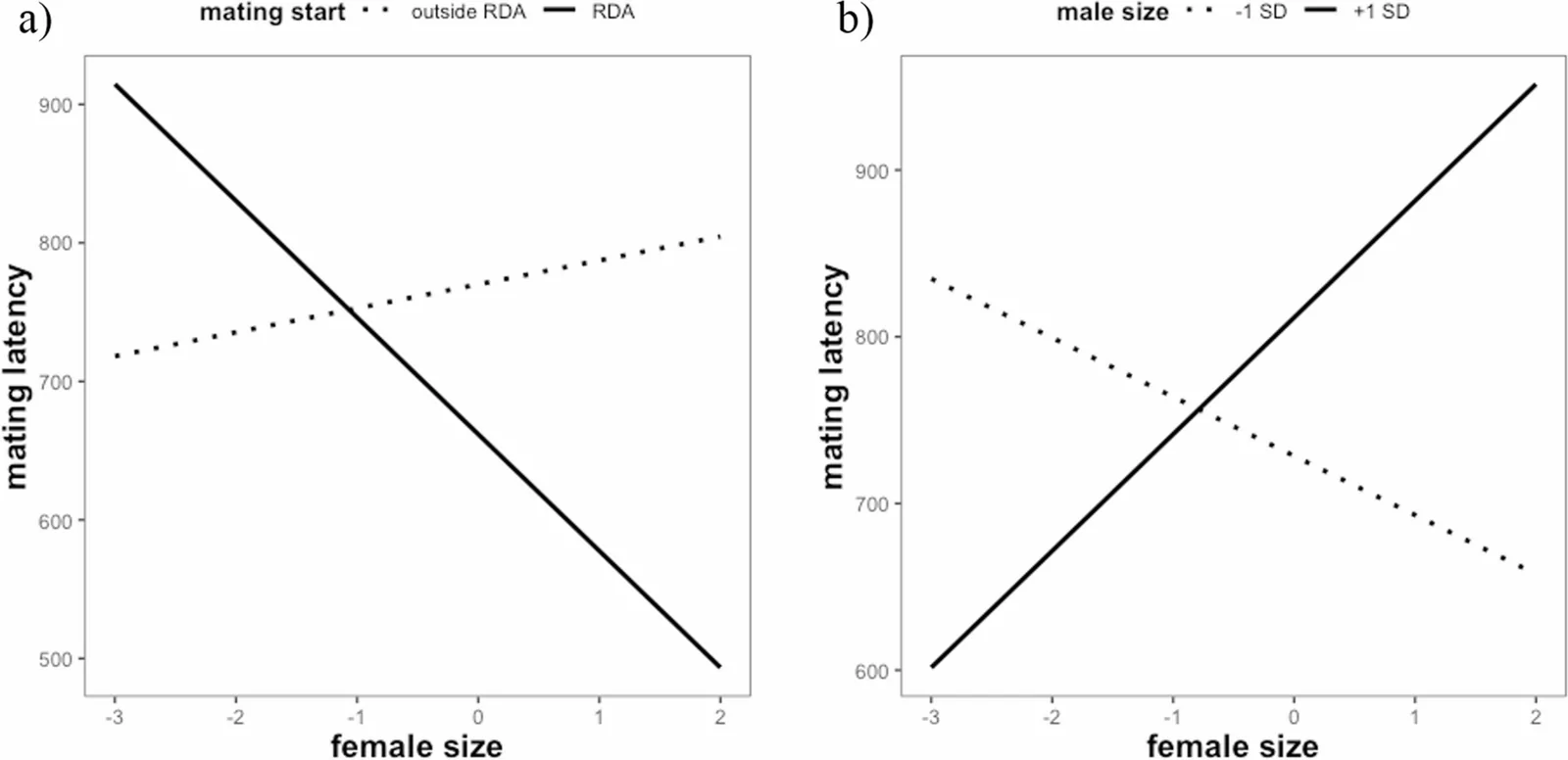
Predicted mating latency depending on female size and (**a**) the location at the start of the mating (solid: in RDA, dashed outside of RDA) and (**b**) male size (solid: male size = mean + 1 standard deviation (SD); dashed: male size = mean – 1 SD)

Successful matings (*n* = 232 across 40 arenas) showed a tendency for size-disassortative mating, in that small females were more likely to mate with large males and vice versa (*t* = −1.75, *P* = 0.082; Table S16). In addition, large females had a marginally higher probability of starting copulation in an RDA (GLMM: *z* = 1.80, *P =* 0.072; Table S15), with no effect of male size (*z* = 1.32, *P* = 0.19). The time to a female’s first mating (*n* = 229) showed an interaction between female size and where it started: matings initiated in RDAs tended to start sooner for large females, but later for small females, than those observed outside these areas (*X*^2^_1_ = 3.64, *P =* 0.056; Fig. 2a; Table S16). Additionally, large females tended to mate later with large males, while small females showed the opposite trend (*X*^2^_1_ = 3.15, *P =* 0.076; Fig. 2b). However, a separate model testing female size alone revealed no effect on mating latency (*t* = -1.55, *P* = 0.12).

Finally, copulation duration depended on an interaction between the starting location and either male (*X*^2^_1_ = 8.33, *P =* 0.004; Fig. S12; Table S17) or female body size (*X*^2^_1_ = 4.12, *P =* 0.043). Specifically, for matings beginning in an RDA, body size of either sex did not affect duration, but matings initiated elsewhere were shorter when involving large males or females. However, copulation duration decreased with increasing time spent in RDAs during copulation (*t* = -2.11, *P* = 0.036) but increased with male courtship in the minute preceding mating (*t* = 3.20, *P* = 0.002).

## Discussion

Our findings highlight how resource distribution shapes condition-dependent reproductive tactics in *Drosophila prolongata*. Large males were more aggressive and increased access to matings through resource occupancy and courtship, whilst small males gained matings through enhanced mobility despite spending comparable amounts of time in RDAs. Similarly, large unmated females spent more time in large RDAs than small females, which increased their probability of mating. Large females were also more likely to initiate a mating, and did so faster, in an RDA than outside, whilst small females showed delayed mating in RDAs relative to the periphery. Unexpectedly, we observed a tendency for size-disassortative mating, with smaller females more likely to mate with relatively large males and vice versa.

### Condition-dependent male mating tactics

Our results corroborate De Nardo et al. (2025a) findings of male *D. prolongata* enhancing their mating success by occupying and defending RDAs. However, decision-making regarding territoriality can also be modulated by (i) individual male condition, (ii) the economic defensibility of the RDA (Brown 1964; Grant 1993), (iii) female preference for specific sites and (iv) the anticipated fitness benefits, each of which we will discuss in turn.

Regarding male condition, we found no effect of body size on male territoriality, even though body size is a common proxy for condition in insects due to its sensitivity to developmental environments (e.g., Partridge et al. 1994). This lack of a relationship may be explained at least in part by the focus of our analyses on the initial 30 min of interactions among newly introduced individuals. Monitoring five consecutive days, De Nardo et al. (2025a) reported no effect of body size on RDA occupancy on the first day, but that larger males exhibit higher territorial success thereafter, possibly due to higher stamina. The establishment of territorial hierarchies within a novel environment is a temporal process that may be protracted under conditions of intense male competition and become increasingly complex with multiple potential territories (Yurkovic et al. 2006; Amino and Matsuo 2023).

Finally, we found that males in RDAs had higher mating success, particularly those occupying the large or both RDAs. Although likely more energetically costly to defend, large RDAs were the preferred mating locations (Mukherjee et al. 2026), thereby enhancing fitness but also serving as a primary driver for male-male competition. Beyond RDA monopolization, courtship significantly increased relative mating success and was observed at a higher rate for males within RDAs, particularly those with high occupancy rates.

While our analysis identified occupancy-L and courtship as the principal predictors of overall mating success, this pattern did not uniformly apply across all males, in that small and large males appeared to gain mating success through different tactics. Specifically, large but not small males gained a greater mating advantage through RDA occupancy, even though body size did not directly correlate with time spent in RDAs. Similarly, despite the absence of a direct link between body size and courtship rate, courtship enhanced mating success for large but not for small males. Instead, the latter achieved mating success through increased movement, supporting a scramble competition tactic (Herberstein et al. 2017). Additionally, aggressive behavior constituted a higher proportion of the total time for large compared to small males. Although aggression positively correlated with RDA occupancy, we found no direct effect of body size on occupancy, suggesting that aggression was likely more critical for territorial success than size during this early phase of hierarchy establishment. Yet, small males may lack the sustained energetic capacity to maintain the necessary level of aggression for prolonged competition (e.g.Amino and Matsuo 2023; De Nardo et al. 2025a). Overall, aggression increased mating success, mediated by occupancy-L but only small males derived a marginal benefit from additional aggression. This suggests that small males exhibiting unexpectedly high aggression for their size might signal superior quality, potentially increasing their attractiveness to females. This might be the case particularly before the establishment of territorial hierarchies (Yurkovic et al. 2006), when small males still have the stamina for competition and when female receptivity is high (i.e., resource value is higher; Hack et al. 1997; Guerra and Mason 2005). Alternatively, the benefit of aggression for small males might be linked to its role in scramble competition. While not inherently an aggressive tactic, males engaged in scrambling for access to receptive females sometimes form multi-male aggregations (Herberstein et al. 2017; De Nardo et al. 2025a), during which aggressive behaviors such as lunging or head butts are occasionally observed (personal observation). Conversely, large males experienced a reduction in mating success when aggression was overexpressed relative to its effect on occupancy. Given the mutually exclusive nature of aggression and courtship, overly aggressive large males may forfeit opportunities to engage in courtship, a behavior that appears to yield greater mating benefits for them.

### Condition-dependent female mating tactics

Based on the premise that mate choice incurs inherent costs, we predicted condition-dependent variation in female mating strategies. Specifically, we hypothesized two potential scenarios: either large females would exhibit a stronger preference for large, territorial males, or small females would adopt an opportunistic strategy by simply mating with males present in an RDA. While these two options might appear mutually exclusive, our findings revealed a more nuanced pattern. We observed that large, rather than small females displayed a stronger propensity for mating within RDAs, in part by spending more time in large RDAs, where matings were most likely to start. Additionally, copulations involving larger females occurred earlier if initiated within RDAs, whereas small females mated faster outside of RDAs. Intriguingly, despite the territorial mating preferences exhibited by large females, we found evidence of size-disassortative mating: large females were more likely to mate, and mated sooner, with small males, whilst small females showed the opposite patterns. These condition-dependent differences may reflect distinct cost-benefit negotiations for females of varying size, although their interpretation remains somewhat speculative, as discussed across the following three non-mutually exclusive potential explanations.

First, unmated females in high condition may spend more time in RDAs, potentially due to their higher tolerance to male harassment (MacPherson et al. 2018; Rostant et al. 2020) or a reduced risk of injury near males engaging in territorial disputes. However, given that male hierarchies are still in the process of establishment during this early phase of our observations (Yurkovic et al. 2006; Amino and Matsuo 2023; De Nardo et al. 2025a), mating with males within territories did not guarantee a large mate. As females are likely unable to ascertain whether male hierarchies have stabilized, they may mate indiscriminately in territories, a behavior that may only favor large males later in the process of settling hierarchies (De Nardo et al. 2025a). Additionally, large *D. prolongata* females exhibit a higher mating rate (De Nardo et al. 2025a) and might initially be less choosy to ensure fertilization (Kokko and Mappes 2005). Even if the initial mate is small, they may mate with larger males in subsequent encounters (Gabor and Halliday 1997; Bateman et al. 2001; Pitcher et al. 2003; Caspers et al. 2014; De Nardo et al. 2025a). If large females experience less harm from males (Harano et al. 2012; Turiegano et al. 2013; MacPherson et al. 2018; Verma et al. 2024), for example by being less vulnerable to physical injury during copulation (e.g., Schäfer and Uhl 2005; Harano et al. 2012) or less susceptible to manipulation by seminal fluid proteins (e.g., Wigby et al. 2016; De Nardo et al. 2021), this initial mating may not impose a significant cost, particularly with a small male (Pitnick and García–González 2002).

Secondly, large, highly fecund females may attract more courtship attention (Wong and Svensson 2009; Kahn et al. 2013; Turiegano et al. 2013) and be followed by multiple males. The attraction of numerous males may lead to scramble competition for access to the female, or males employing a scrambling tactic may be generally more attuned and responsive to cues of female receptivity (Herberstein et al. 2017). Furthermore, scrambling males may be particularly attentive to females within RDAs if this context tends to signal receptiveness. This could potentially explain the higher mating success of small males with large females, as scramble competition often favors smaller males (Herberstein et al. 2017; De Nardo et al. 2025a; Nardo et al. 2025b phenomenon that would be especially pronounced if large females were less discriminatively receptive in territories.

Third, small *D. prolongata* females exhibit a lower mating rate (De Nardo et al. 2025a), possibly due to their higher cost per mating (Harano et al. 2012). Similarly, they may be more vulnerable to male harassment and injury (MacPherson et al. 2018; Rostant et al. 2020) and may therefore avoid territories, especially in the initial stages. Due to their lower mating rate, they have fewer opportunities for trading-up, and if matings were more costly, they would have a higher threshold for mate acceptance (Frame and Mills 2014). While the effect was not statistically significant, small females tended to initiate copulations later, which could indicate longer decision-making processes. Alternatively, as small females avoid territories, they may mate with the first acceptable courter encountered, potentially selecting for larger males (De Nardo et al. 2025a).

To gain a more comprehensive understanding of female choice strategies, future research could investigate how females varying in condition and mating status, adjust their rejection and mate assessment behaviors, as well as their preferred mating locations, under diverse socio-ecological contexts.

### Concurrent mating systems and female choice

In species with flexible ARTs the primary tactic often leads to higher reproductive success compared to the alternative tactic (Da Silva et al. 2025). Given the higher mating success of large males overall and the competition for territory occupancy across all size classes, resource defense is likely the primary mating system in *D. prolongata*. This inference is supported by the male-biased sexual size dimorphism, male aggressiveness, and the presence of enlarged forelegs used in intrasexual combats (Kudo et al. 2015). However, accumulating evidence suggests that alternative mating strategies can be expressed and prove more advantageous depending on the ecological context and individual condition. For example, De Nardo et al. (2025a) show that body size increases mating success more at higher compared to lower fly densities when RDAs can be monopolized, whereas small males gain higher mating success than large males in experimental setups with a uniform food distribution that limit territorial behavior (*t* = -2.25, *n* = 192, *P* = 0.035, Perdigón Ferreira & Lüpold, unpublished; Muro 2021). Moreover, larger and more territorial males achieve higher reproductive success under an even operational sex ratio (OSR), but not under male-biased OSRs (De Nardo et al. 2025a). This pattern is further reinforced by the finding that males from populations evolving under a male-biased OSR are significantly smaller than those evolving under an even OSR (De Nardo et al. 2025b).

Here, we found that a territorial strategy was successful for large males, whereas a scramble competition strategy conferred benefits exclusively to small males. Scramble competition represents a prevalent mating system among invertebrates, typically favoring traits such as mobility, agility, and the ability to locate receptive mates rapidly (Herberstein et al. 2017), where morphological traits such as small body size and relatively larger locomotory appendages can be advantageous (e.g., Ghiselin 1974; Blanckenhorn et al. 1995; Able 1999; Moya-Laraño et al. 2007; Kelly et al. 2008; Herberstein et al. 2017; Kelly 2020). However, as our findings suggest, the expression of scramble competition can depend on the environmental or social context and on individual condition (McLain 1992; Von Kuerthy and Taborsky 2016; Herberstein et al. 2017). Where it is not the primary tactic, alternative reproductive tactics (ARTs) can manifest as a form of scramble competition (Von Kuerthy and Taborsky 2016; Herberstein et al. 2017). Male *D. prolongata* show a distinct ART involving the interception of a female while another male is stimulating her abdomen with leg vibrations, an important component of courtship in this species (Minekawa et al. 2018; Perdigón Ferreira and Lüpold 2022). Such interception behavior has been shown to depend on individual condition and the social context (Perdigón Ferreira and Lüpold 2022). As this ART is more common among small males (Perdigón Ferreira and Lüpold 2022; De Nardo et al. 2025a) and requires speed and agility, it likely represents an important, albeit not exhaustive component of the scramble competition tactic in this species.

Beyond interception, we propose that other behaviors may constitute elements of a scramble competition tactic in small *D. prolongata* males: (1) spending time in territories during periods of hierarchy establishment or large-male engagement in copulation or courtship could pay off, particularly if females within territories are less discriminatory; (2) promptly approaching and following receptive females both in the peripheral areas and within territories, consistent with our observation of increased mating success in highly mobile small males, with no corresponding effect of courtship; (3) attempting sneak copulations from courting males in the absence of leg vibration, a behavior that has previously been observed in over 65% of 156 alternative mating events (De Nardo et al. 2025a).

Speed and mobility are critical traits in scramble competition mating systems. With the first male to reach a receptive female typically securing the mating (Herberstein et al. 2017), scramble competition may exert selective pressure for reduced female choosiness (Dechaume-Moncharmont et al. 2016). However, if female choice is constrained, the persistent higher mating success of large territorial males requires explanation. As previously discussed, female choice is costly (Parker 1983; Lima and Dill 1990; Dale et al. 1992; Byers et al. 2005; Lindström and Lehtonen 2013), but the associated costs and benefits can vary with female condition (Hunt et al. 2005; Frame and Mills 2014). One evolutionarily stable mate choice strategy with minimal costs would be choosing males indirectly by their location (e.g., lek or territory; Wiley and Poston 1996; Castellano 2009; Kovalov and Kokko 2022). While this form of mate choice can select for high stamina in males, it can also facilitate the evolution of ARTs in low-condition males (Castellano 2009; Kovalov and Kokko 2022). Similarly, large *D. prolongata* females prefer mating in territories but do not appear to exhibit a preference for larger males. Over extended periods, this would tend to favor large, territorial males (De Nardo et al. 2025a), but continued acceptance of smaller males would also allow scramble competition tactics to persist within the same population (Lanctot and Weatherhead 1997; Bro-Jørgensen 2011). This suggests that relying on an indirect cue for mate quality (Wiley and Poston 1996), such as the size or quality of a local resource, could enable low-condition males to gain reproductive success through alternative tactics. While minimizing the female’s cost of rejection (Harano 2015), it would also allow for the differential success of male tactics depending on the prevailing context.

If at least some females employ indirect choice, context-dependent shifts in the dominant mating system become more readily explicable. For example, under a male-biased OSR, scramble competition becomes the more successful mating tactic in *D. prolongata* (De Nardo et al. 2025a; Nardo et al. 2025b), likely due to the reduced efficacy of territorial defense at high competitor densities (Grant 1993; McLain 1992; Lüpold et al. 2017; McCullough et al. 2018; De Nardo et al. 2025a). This shift can be understood by the inherent presence of some scrambling males in any population, without necessitating a change in female preference. Moreover, given the high remating rate in this species (De Nardo et al. 2025a), post-copulatory sexual selection plays a significant role, favoring high-condition males (De Nardo et al. 2025b). This could further mitigate the cost of opportunistically mating with a lower-quality male, as a female’s subsequent mates may be larger and more successful in sperm competition (De Nardo et al. 2025a).

## Conclusions

While our study does not provide clear evidence for passive female choice, several indications suggest that females may use resource-dense areas as cues for mate choice, potentially contingent on their condition, rather than directly favoring high-condition mates. This could provide a mechanism for the observed coexistence of resource defense and scramble competition in this species. Similarly, it is plausible that multiple female mating tactics co-occur, although our understanding of the nuances differentiating these tactics remains limited. Collectively, these findings contribute to our broader understanding of the mechanisms maintaining genetic variation in populations subject to sexual selection (Rowe and Houle 1996). Our speculation on the potential role of indirect female choice in this species is intended to motivate further research into how divergent female reproductive tactivs are maintained within a single species in response to condition and socio-ecological context, thereby improving our understanding of how natural populations evolve under fluctuating environments.

## Supplementary information

Below is the link to the electronic supplementary material.

Supplementary File 1 (DOCX 5.70 MB)

## Data Availability

The datasets are available as supplementary material.
