# Supplementary material for: Condition-dependent success of resource-based reproductive tactics across both sexes in Drosophila prolongata
Source: Behav Ecol Sociobiol. 2026 May 7;80(5):61. doi: 10.1007/s00265-026-03739-z (PMC13658425; doi:10.1007/s00265-026-03739-z)
Supplement: Supplementary file 1 [file 265_2026_3739_MOESM1_ESM.docx]

**Supplementary Information**

for

**“Condition-dependent success of resource-based reproductive tactics across both sexes in *Drosophila prolongata”***

Alessio N. De Nardo, Swarnima Mukherjee & Stefan Lüpold

**Supplementary Figures**

**B**

**A**

**Figure S1:** The left image shows an example of an experimental unit, in this case with a small (A) and large (B) resource-dense area (RDA) of high quality. The right image shows the same experimental unit, with the regions of interest (ROIs) overlayed on the RDAs. The red areas mark the RDAs, whereas the green area marks the buffer zone added around the small RDA, to enhance comparability of their occupancy.

**Figure S2:** Distribution of body sizes (thorax lengths in mm) of males (blue) and females (red) per arena.

**Figure S3:** Distribution of male body sizes (thorax lengths in mm) and categorization into small (green) and large (blue) by clustering approach within each arena.

**Figure S4:** Distribution of thorax length depending on sex and developmental food quality (raw data and density). The predicted means ± 95% confidence intervals are shown in black.

**a)**

**b)**

**c)**

low

high

outside

small RDA

large RDA

outside

small RDA

large RDA

**Figure 5:**

**Figure S5: a)** While the greatest share of male aggression time was observed outside RDAs, it was much lower than expected by chance (dashed line). In contrast, more aggression than expected by chance was observed in the small and large RDAs. **b)** Males spent a larger proportion of their time in the small and large RDAs on aggression compared to their time spent on aggression outside the RDAs. **c)** The high proportion of aggression shown outside of RDAs was largely expressed by males with very low occupancy of the large RDA (Occupancy-L). Males with high occupancy of the large RDA (occupancy-L) and high occupancy of the small RDA (occupancy-S) showed more aggression outside of the RDAs compared to those with low occupancy of the small RDA. The black points and error bars in plots **a)** and **b)** represent the mean ± 95% confidence limits of the means.

**a)**

**b)**

**c)**

low

high

outside

small RDA

large RDA

outside

small RDA

large RDA

**Figure S6: a)** While the greatest share of total courtship time was observed outside RDAs, it was much lower than expected by chance (dashed line). In contrast, more courtship than expected by chance was observed in the small and large RDAs. **b)** Males spent a larger proportion of their time on courtship in the small and large RDAs compared to their time spent on courtship outside the RDAs. **c)** The high proportion of courtship shown outside of RDAs was largely expressed by males with very low occupancy of the large RDA (Occupancy-L). Males with high occupancy of the large RDA (Occupancy-L) and high occupancy of the small RDA (Occupancy-S) showed more aggression outside of the RDAs compared to those with low occupancy of the small RDA. The black points and error bars in plots **a)** and **b)** represent the mean ± 95% confidence limits of the means.

**Figure S7:** Courtship increased with occupancy of the large RDA (occupancy-L), more so in large males (solid: male size = mean + 1 SD) rather than small males (dashed: male size = mean – 1 SD).

**Figure S8:** Relative mating success decreased when large males (solid: male size = mean + 1 SD) were overly aggressive but slightly increased it for small males (dashed: male size = mean – SD).

**Figure S9:** Relative mating success was affected by occupancy of the large RDA (occupancy-L) especially in large males (solid: male size = mean + 1 SD) compared to small males (dashed: male size = mean – SD), if they also had average-to-high occupancy of the small RDA (occupancy-S).

**Figure S10:** Visualization of the structural equation model across all males. Green arrows indicate significantly positive effects, red arrows indicate significantly negative effects, while grey arrows indicate hypothesized effects with P > 0.05. The width of the arrow reflects the effect size.

**Figure S11:** Visualization of the structural equation model in females. Green arrows indicate significantly positive effects, red arrows indicate significantly negative effects, while grey arrows indicate hypothesized effects with P > 0.05. The width of the arrow reflects the effect size.

**Figure S12:** Copulation duration (a) decreased with female size if the mating started outside the RDAs and (b) decreased with male size if the mating started outside the RDAs. Solid: mating start in RDA, dashed: mating start outside RDA.

**a)**

**b)**

**Supplementary Tables**

**Table S1:** Ethogram of male aggression behaviors, which was followed for labeling the training data for categorization of aggression

| **Behavior** | **Definition** |
| --- | --- |
| Lunging | Rearing up and collapsing onto opponent |
| Chasing | Running after opponent |
| Head butt | Thrusting head into opponents body |
| Tussling | Tumbling over opponent |
| Fencing | Pushing opponents leg |
| Boxing | Striking/shoving opponent with forelegs while both are reared on hindlegs |

**Table S2a and b:** ANOVA (a) and summary table (b) on the thorax length in mm. Significant *P*-values are in bold and trends (*P* < 0.1) are in italics.

**a)**

|  | ***X²*** | ***df*** | ***P*** |
| --- | --- | --- | --- |
| **Food treatment** | 194.39 | 2 | **< 0.001** |
| **Sex** | 1272.30 | 1 | **< 0.001** |
| **Food treatment × Sex** | 4.93 | 2 | *0.085* |

**b)**

|  | ***Est.*** | ***SE*** | ***t*** | ***P*** |
| --- | --- | --- | --- | --- |
| **Intercept** | 1.55 | 0.01 | 212.64 | **< 0.001** |
| **Food treatment (intermediate)** | -0.05 | 0.01 | -4.68 | **< 0.001** |
| **Food treatment (low)** | -0.11 | 0.01 | -8.87 | **< 0.001** |
| **Sex (male)** | 0.22 | 0.01 | 21.75 | **< 0.001** |
| **Food treatment (intermediate) × Sex (male)** | 0.02 | 0.01 | 1.30 | 0.20 |
| **Food treatment (low) × Sex (male)** | -0.02 | 0.02 | -1.08 | 0.28 |

**Table S3:** Summary table on male occupancy of the large RDA (occupancy-L). Significant *P*-values are in bold.

|  | ***Est.*** | ***SE*** | ***t*** | ***P*** |
| --- | --- | --- | --- | --- |
| **Intercept** | 0.00 | 0.05 | 0.00 | 1.00 |
| **Thorax length** | -0.01 | 0.05 | -0.23 | 0.82 |
| **Aggression** | 0.46 | 0.05 | 9.11 | **< 0.001** |

**Table S4:** Summary table on male occupancy of the small RDA (occupancy-S). Significant *P*-values are in bold.

|  | ***Est.*** | ***SE*** | ***t*** | ***P*** |
| --- | --- | --- | --- | --- |
| **Intercept** | 0.00 | 0.05 | 0.00 | 1.00 |
| **Thorax length** | -0.05 | 0.05 | -0.92 | 0.36 |
| **Aggression** | 0.09 | 0.06 | 1.48 | 0.14 |
| **Occupancy-L** | -0.45 | 0.06 | -7.76 | **< 0.001** |

**Table S5:** ANOVA (a) and summary table (b) on the proportion of male aggression outside of RDAs. Significant *P*-values are in bold.

**a)**

|  | ***X²*** | ***df*** | ***P*** |
| --- | --- | --- | --- |
| **Occupancy-L** | 305.29 | 1 | **< 0.001** |
| **Occupancy-S** | 8.58 | 1 | **0.003** |
| **Occupancy-S × Occupancy-L** | 5.96 | 1 | **0.015** |

|  | ***Est.*** | ***SE*** | ***z*** | ***P*** |
| --- | --- | --- | --- | --- |
| **Intercept** | 1.06 | 0.12 | 8.74 | **< 0.001** |
| **Occupancy-L** | -3.36 | 0.26 | -12.95 | **< 0.001** |
| **Occupancy-S** | -0.04 | 0.13 | -0.30 | **0.76** |
| **Occupancy-S × Occupancy-L** | 0.81 | 0.33 | 2.44 | **0.015** |

**b)**

**Table S6:** ANOVA (a) and summary table (b) on courtship. Significant *P*-values are in bold.

**a)**

|  | ***X²*** | ***df*** | ***P*** |
| --- | --- | --- | --- |
| **Thorax length** | 0.19 | 1 | 0.66 |
| **Occupancy-L** | 29.19 | 1 | **< 0.001** |
| **Occupancy-S** | 8.83 | 1 | **0.003** |
| **Thorax length × Occupancy-L** | 5.94 | 1 | **0.015** |

**b)**

|  | ***Est.*** | ***SE*** | ***t*** | ***P*** |
| --- | --- | --- | --- | --- |
| **Intercept** | 0.00 | 0.05 | -0.11 | 0.92 |
| **Thorax length** | 0.04 | 0.05 | 0.76 | 0.45 |
| **Occupancy-L** | 0.31 | 0.06 | 5.37 | **< 0.001** |
| **Occupancy-S** | 0.17 | 0.06 | 2.97 | **0.003** |
| **Thorax length × Occupancy-L** | 0.14 | 0.06 | 2.44 | **0.015** |

**Table S7:** ANOVA (a) and summary table (b) on mobility. Significant *P*-values are in bold.

**a)**

|  | ***X²*** | ***df*** | ***P*** |
| --- | --- | --- | --- |
| **Thorax length** | 2.61 | 1 | 0.11 |
| **Occupancy-L** | 8.82 | 1 | **0.003** |
| **Occupancy-S** | 1.45 | 1 | 0.23 |
| **Aggression** | 86.48 | 1 | **< 0.001** |
| **Courtship** | 322.81 | 1 | **< 0.001** |
| **Thorax length × Aggression** | 5.87 | 1 | **0.015** |

**b)**

|  | ***Est.*** | ***SE*** | ***t*** | ***P*** |
| --- | --- | --- | --- | --- |
| **Intercept** | -0.01 | 0.03 | -0.30 | 0.76 |
| **Thorax length** | 0.06 | 0.03 | 2.00 | **0.046** |
| **Occupancy-L** | -0.11 | 0.04 | -2.97 | **0.003** |
| **Occupancy-S** | -0.04 | 0.03 | -1.21 | 0.23 |
| **Aggression** | 0.35 | 0.04 | 9.19 | **< 0.001** |
| **Courtship** | 0.63 | 0.04 | 17.97 | **< 0.001** |
| **Thorax length × Aggression** | 0.08 | 0.03 | 2.42 | **0.016** |

**Table S8:** ANOVA (a) and summary table (b) on relative mating success. Significant *P*-values are in bold, trends (*P* < 0.1) are in italics.

**a)**

|  | ***X²*** | ***df*** | ***P*** |
| --- | --- | --- | --- |
| **Mobility** | 3.98 | 1 | **0.049** |
| **Occupancy-L** | 4.85 | 1 | **0.028** |
| **Thorax length** | 2.38 | 1 | 0.12 |
| **Occupancy-S** | 1.08 | 1 | 0.30 |
| **Aggression** | 0.38 | 1 | 0.54 |
| **Courtship** | 6.68 | 1 | **0.010** |
| **Occupancy-L × Thorax length** | 1.73 | 1 | 0.19 |
| **Occupancy-L × Occupancy-S** | 1.72 | 1 | 0.19 |
| **Thorax length × Occupancy-S** | 2.51 | 1 | 0.11 |
| **Thorax length × Aggression** | 4.02 | 1 | **0.045** |
| **Occupancy-L × Thorax length × Occupancy-S** | 7.15 | 1 | **0.007** |

**b)**

|  | ***Est.*** | ***SE*** | ***t*** | ***P*** |
| --- | --- | --- | --- | --- |
| **Intercept** | 0.132 | 0.01 | 16.35 | **< 0.001** |
| **Mobility** | 0.039 | 0.01 | 2.00 | **0.047** |
| **Occupancy-L** | 0.025 | 0.01 | 2.45 | **0.015** |
| **Thorax length** | 0.018 | 0.01 | 2.05 | **0.042** |
| **Occupancy-S** | 0.015 | 0.01 | 1.57 | 0.12 |
| **Aggression** | -0.007 | 0.01 | -0.63 | 0.53 |
| **Courtship** | 0.034 | 0.01 | 2.56 | **0.010** |
| **Occupancy-L × Thorax length** | 0.018 | 0.01 | 1.72 | *0.087* |
| **Occupancy-L × Occupancy-S** | 0.009 | 0.01 | 1.04 | 0.30 |
| **Thorax length × Occupancy-S** | 0.027 | 0.01 | 2.73 | **0.007** |
| **Thorax length × Aggression** | -0.020 | 0.01 | -2.01 | **0.046** |
| **Occupancy-L × Thorax length × Occupancy-S** | 0.027 | 0.01 | 2.67 | **0.008** |

**Table S9:** ANOVA (a) and summary table (b) on relative mating success in large males. Significant *P*-values are in bold.

**a)**

|  | ***X²*** | ***df*** | ***P*** |
| --- | --- | --- | --- |
| **Occupancy-L** | 4.36 | 1 | **0.037** |
| **Occupancy-S** | 1.72 | 1 | 0.19 |
| **Courtship** | 6.97 | 1 | **0.008** |
| **Thorax length** | 0.06 | 1 | 0.80 |
| **Aggression** | 2.07 | 1 | 0.15 |
| **Mobility** | 0.45 | 1 | 0.50 |
| **Occupancy-L × Occupancy-S** | 4.08 | 1 | **0.043** |

**b)**

|  | ***Est.*** | ***SE*** | ***t*** | ***P*** |
| --- | --- | --- | --- | --- |
| **Intercept** | 0.15 | 0.02 | 8.88 | < 0.001 |
| **Occupancy-L** | 0.03 | 0.01 | 2.47 | **0.014** |
| **Occupancy-S** | 0.03 | 0.01 | 2.08 | **0.039** |
| **Courtship** | 0.05 | 0.02 | 2.64 | **0.009** |
| **Thorax length** | -0.01 | 0.02 | -0.25 | 0.80 |
| **Aggression** | -0.02 | 0.02 | -1.44 | 0.15 |
| **Mobility** | 0.01 | 0.02 | 0.67 | 0.50 |
| **Occupancy-L × Occupancy-S** | 0.03 | 0.01 | 2.02 | **0.045** |

**Table S10:** ANOVA (a) and summary table (b) on relative mating success in small males. Significant *P*-values are in bold, trends (*P* < 0.1) in italics

**a)**

|  | ***X²*** | ***df*** | ***P*** |
| --- | --- | --- | --- |
| **Occupancy-S** | 0.02 | 1 | 0.88 |
| **Occupancy-L** | 0.64 | 1 | 0.42 |
| **Courtship** | 0.20 | 1 | 0.65 |
| **Thorax length** | 3.60 | 1 | *0.057* |
| **Aggression** | 0.59 | 1 | 0.44 |
| **Mobility** | 9.84 | 1 | **0.002** |

**b)**

|  | ***Est.*** | ***SE*** | ***t*** | ***P*** |
| --- | --- | --- | --- | --- |
| **Intercept** | 0.15 | 0.02 | 8.69 | **< 0.001** |
| **Occupancy-S** | 0.00 | 0.01 | 0.16 | 0.88 |
| **Occupancy-L** | 0.01 | 0.01 | 0.80 | 0.43 |
| **Courtship** | 0.01 | 0.02 | 0.45 | 0.65 |
| **Thorax length** | 0.03 | 0.02 | 1.90 | *0.060* |
| **Aggression** | 0.01 | 0.02 | 0.77 | 0.44 |
| **Mobility** | 0.07 | 0.02 | 3.14 | **0.002** |

**Table S11:** Summary table on female mating probability. Significant *P*-values are in bold

|  | ***Est.*** | ***SE*** | ***z*** | ***P*** |
| --- | --- | --- | --- | --- |
| **Intercept** | 1.04 | 0.16 | 6.46 | **< 0.001** |
| **Thorax length** | 0.10 | 0.14 | 0.73 | 0.46 |
| **Occupancy-L (as virgin)** | 0.48 | 0.17 | 2.83 | **0.005** |
| **Occupancy-S (as virgin)** | 0.16 | 0.15 | 1.11 | 0.27 |
| **Mobility (as virgin)** | 0.15 | 0.15 | 1.01 | 0.31 |

**Table S12:** Summary table on female occupancy-L as virgin. Significant *P*-values are in bold

|  | ***Est.*** | ***SE*** | ***t*** | ***P*** |
| --- | --- | --- | --- | --- |
| **Intercept** | 0.00 | 0.05 | 0.00 | 1.00 |
| **Thorax length** | 0.14 | 0.05 | 2.51 | **0.013** |
| **Mobility** | 0.19 | 0.05 | 3.46 | **< 0.001** |

**Table S13:** Summary table on female occupancy-S as virgin. Significant *P*-values are in bold

|  | ***Est.*** | ***SE*** | ***t*** | ***P*** |
| --- | --- | --- | --- | --- |
| **Intercept** | 0.00 | 0.05 | 0.00 | 1.00 |
| **Thorax length** | 0.03 | 0.06 | 0.45 | 0.65 |
| **Occupancy-L (as virgin)** | -0.24 | 0.06 | -4.18 | **< 0.001** |
| **Mobility** | 0.09 | 0.06 | 1.57 | 0.12 |

**Table S14:** Standard estimates (Std. est.) and *P*-values of structural equation model in females. Significant estimates are indicated in bold (*P* < 0.05).

| **Response** | **Predictor** | **Std. est.** | ***P*** |
| --- | --- | --- | --- |
| **Mating success** | **Body size** | 0.05 | 0.46 |
|  | **Occupancy-L** | **0.24** | **0.005** |
|  | **Occupancy-S** | 0.08 | 0.27 |
|  | **Mobility** | 0.07 | 0.31 |
| **Occupancy-L** | **Body size** | **0.14** | **0.013** |
|  | **Mobility** | **0.19** | **< 0.001** |
| **Occupancy-S** | **Body size** | 0.03 | 0.65 |
|  | **Occupancy-L** | **-0.24** | **< 0.001** |
|  | **Mobility** | 0.09 | 0.12 |
| **Mobility** | **Body size** | -0.06 | 0.31 |

**Table S15:** Summary table on the probability of matings starting in an RDA. Significant *P*-values are in bold, trends (*P* < 0.1) in italics

|  | ***Est.*** | ***SE*** | ***z*** | ***P*** |
| --- | --- | --- | --- | --- |
| **Intercept** | -7.61 | 4.00 | -1.89 | *0.058* |
| **Female thorax length** | 0.77 | 0.43 | 1.80 | *0.072* |
| **Male thorax length** | 0.56 | 0.42 | 1.32 | 0.19 |

**Table S16:** ANOVA (a) and summary table (b) on mating latencies. Significant *P*-values are in bold, trends (*P* < 0.1) in italics

**a)**

|  | ***X²*** | ***df*** | ***P*** |
| --- | --- | --- | --- |
| **Female thorax length** | 1.41 | 1 | 0.23 |
| **Male thorax length** | 3.15 | 1 | *0.076* |
| **Start in large RDA** | 3.68 | 1 | *0.055* |
| **Start in small RDA** | 0.22 | 1 | 0.64 |
| **Female thorax length × Male thorax length** | 3.15 | 1 | *0.076* |
| **Female thorax length × Start in large RDA** | 3.70 | 1 | *0.054* |

**b)**

|  | ***Est.*** | ***SE*** | ***t*** | ***P*** |
| --- | --- | --- | --- | --- |
| **Intercept** | 0.20 | 0.12 | 1.67 | *0.098* |
| **Female thorax length** | 0.05 | 0.09 | 0.49 | 0.62 |
| **Male thorax length** | 0.10 | 0.07 | 1.50 | 0.13 |
| **Start in large RDA** | -0.29 | 0.14 | -2.00 | **0.048** |
| **Start in small RDA** | -0.10 | 0.21 | -0.47 | 0.64 |
| **Female thorax length × Male thorax length** | 0.13 | 0.07 | 1.78 | *0.077* |
| **Female thorax length × Start in large RDA (yes)** | -0.25 | 0.13 | -1.92 | *0.056* |

**Table S17:** ANOVA (a) and summary table (b) on mating duration. Significant *P*-values are in bold, trends (*P* < 0.1) in italics

**a)**

|  | ***X²*** | ***df*** | ***P*** |
| --- | --- | --- | --- |
| **Male thorax length** | 0.99 | 1 | 0.32 |
| **Mating start in RDA** | 1.47 | 1 | 0.22 |
| **Female thorax length** | 0.57 | 1 | 0.45 |
| **RDA Occupancy during mating** | 5.54 | 1 | **0.019** |
| **Courtship before mating** | 10.22 | 1 | **0.001** |
| **Male thorax length × Mating start in RDA** | 8.33 | 1 | **0.004** |
| **Female thorax length × Mating start in RDA** | 4.12 | 1 | **0.043** |

**b)**

|  | ***Est.*** | ***SE*** | ***t*** | ***P*** |
| --- | --- | --- | --- | --- |
| **Intercept** | 0.15 | 0.12 | 1.29 | 0.20 |
| **Male thorax length** | -0.36 | 0.12 | -2.85 | **0.005** |
| **Mating start in RDA** | -0.23 | 0.15 | -1.59 | 0.11 |
| **Female thorax length** | -0.25 | 0.12 | -2.11 | **0.036** |
| **RDA Occupancy during mating** | -0.16 | 0.07 | -2.35 | **0.019** |
| **Courtship before mating** | 0.20 | 0.06 | 3.20 | **0.002** |
| **Male thorax length × Mating start in RDA** | 0.45 | 0.15 | 2.89 | **0.004** |
| **Female thorax length × Mating start in RDA** | 0.29 | 0.14 | 2.03 | **0.044** |
